# In the eye of the beholder: Is color classification consistent among human observers?

**DOI:** 10.1002/ece3.8093

**Published:** 2021-09-14

**Authors:** Kim Valenta, Sally L. Bornbusch, Yan‐Daniel Jacques, Omer Nevo

**Affiliations:** ^1^ Department of Anthropology University of Florida Gainesville FL USA; ^2^ Department of Evolutionary Anthropology Duke University Durham NC USA; ^3^ The Mad Dog Initiative Antananarivo Madagascar; ^4^ German Centre for Integrative Biodiversity Research (iDiv) Halle‐Jena‐Leipzig Leipzig Germany; ^5^ Institute of Biodiversity, Friedrich Schiller University Jena Jena Germany

**Keywords:** classification, color vision, fruits, language, perception, synthesis

## Abstract

Colorful displays have evolved in multiple plant and animal species as signals to mutualists, antagonists, competitors, mates, and other potential receivers. Studies of color have long relied on subjective classifications of color by human observers. However, humans have a limited ability to perceive color compared to other animals, and human biological, cultural, and environmental variables can influence color perception. Here, we test the consistency of human color classification using fruit color as a model system. We used reflectance data of 67 tropical fruits and surveyed 786 participants to assess the degree to which (a) participants of different cultural and linguistic backgrounds agree on color classification of fruits; and (b) human classification to a discrete set of commonly used colors (e.g., red, blue, green) corresponds to natural clusters based on light reflectance measures processed through visual systems of other animals. We find that individual humans tend to agree on the colors they attribute to fruits across language groups. However, these colors do not correspond to clearly discernible clusters in di‐ or tetrachromatic visual systems. These results indicate that subjective color categorizations tend to be consistent among observers and can be used for large synthetic studies, but also that they do not fully reflect natural categories that are relevant to animal observers.

## Graphical abstract

Subjective human color classifications continue to be used regularly in assigning colors to organisms of interest. Here, we investigate whether or not subjective human color classifications are consistent among human observers, and whether intraspecific variation in color assignation can affect study results.

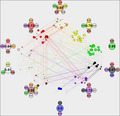

## 1 INTRODUCTION

Colorful displays have evolved in multiple plant and animal species as a means of communicating information to con‐ and heterospecifics (Osorio & Vorobyev, 2008). Intraspecific color signaling is common, particularly in the context of signaling the value of potential mates (Bennett et al., 1994; Dixson et al., 2005; Dubuc et al., 2014), a phenomenon of interest since at least Darwin's time (Darwin, 1896). For interspecific color signaling, an extensive body of literature exists on the importance of color signaling to antagonists, such as conspicuous color as a signal of toxicity to predators, across marine and terrestrial systems (Marples et al., 2005; Ritson‐Williams & Paul, 2007), and particularly among reptiles and amphibians (Maan & Cummings, 2012; Ruxton et al., 2019). Many organisms also rely on cryptic coloration to avoid predation by blending in with surrounding colors (Stuart‐Fox et al., 2004). The importance of color signals to interspecific mutualists has long attracted the attention of biologists and has been particularly well documented for the wide variety of wild flowers and fruit colors and their role in signaling ripeness to pollinating and seed‐dispersing animals (Allen, 1879; van der Pijl, 1969; Renoult et al., 2014; Valenta et al., 2017).

Prior to and following the advent of spectroscopic advances in color quantification, many studies on the ecological and evolutionary relevance of color relied on subjective, human categorizations of color (Brodie, 2017; Burns et al., 2009; Lu et al., 2019; Onstein et al., 2019, 2020; Sinnott‐Armstrong et al., 2018) (Bennett et al., 1994; Endler, 1990; Valenta et al., 2018; Vorobyev & Osorio, 1998). Critiques of these approaches note that most animals do not share human color vision phenotypes, and therefore, these assessments are at best unreliable, and at worst irrelevant (Cronin et al., 2014; Kemp et al., 2015; Valenta et al., 2018). The majority of humans are trichromats, possessing three different types of cones. The human trichromacy phenotype is exceedingly rare across the mammalian Class and is shared only with our closest relatives, the diurnal catarrhines of the Order Primates (Jacobs, 2008a). Most other mammals are dichromatic, making them unable to chromatically distinguish between greens and reds (Jacobs, 2008b), while most birds are able to discern color across a wider range of the spectrum than mammals (Osorio & Vorobyev, 2008). Given the limited ability of humans to detect color relative to most diurnal, nonmammalian species, it is nearly guaranteed that humans cannot perceive the full range of color signals in nature and instead can only detect a subset (Bergeron & Fuller, 2018). In many cases, the human perception of color likely differs substantially from the way that color is perceived by the intended signal recipient, whether they be an antagonist or mutualist (Cronin et al., 2014; Ruxton et al., 2019). This is particularly true for color signals that exist outside of human perception limits, for example, in the range of ultraviolet reflectance (~300–400 nm), which humans are incapable of detecting (Honkavaara et al., 2002).

In addition to variation in color vision across the animal kingdom, subjective human assessments of color can be confounded by several factors that are difficult to control for. There is extensive literature on the relationships between language and color perception and categorization (Goldstein et al., 2009; Lindsey & Brown, 2019; Martinovic et al., 2020; Roberson & Hanley, 2007; Thierry et al., 2009; Witzel, 2019). Cross‐linguistic studies have found evidence that color categorization is strongly linked to language (Athanasopoulos et al., 2010) and, within a given language, can be affected by cultural variation (González‐Perilli et al., 2017). This is particularly problematic for studies that integrate subjective color descriptions collected across geographical areas with speakers of different languages (Brodie, 2017; Sinnott‐Armstrong et al., 2018). Further, although most humans are trichromatic, there is compelling and recent behavioral and molecular evidence that some human females are functionally tetrachromatic, with an ability to distinguish chromatic variation beyond what is normally observed in humans (Jordan & Mollon, 2019). More commonly, approximately 2% of human males are thought to lack one of the three cone types permitting trichromacy, and these human dichromats vary in their color perception compared to both trichromats and to other dichromats with different photoreceptor phenotypes (Álvaro et al., 2015). Thus, even among humans, subjective assignments of colors can vary**—**colors may not be consistently described by different observers, due to biological, linguistic, and cultural variation that may drive human color perception and categorization.

In this study, we used a free, publicly available surveying platform to investigate the consistency of human color perception and categorization, using images of wild fruits as a model system. We showed 67 images of wild fruits for which we obtained spectroscopic data to 786 volunteers from across the globe and asked them to classify each fruit as one of ten colors that are commonly used in seed dispersal literature (red, orange, yellow, green, blue, purple, pink, brown, white, and black). We first test to what degree participants agree on color classifications, across the sample and between speakers of different languages. We then test whether human classification corresponds to natural clusters of fruit color in the eyes of nonhuman observers using reflectance spectra of the same fruits to model their quantum catch in the eyes of a dichromatic mammal and a tetrachromatic bird. In both, we first look at the classification to the ten commonly used categories (red, orange, yellow, green, blue, purple, pink, brown, white, and black) and then look at two higher‐level divisions that have proposed ecological or evolutionary significance to fruit ecology: conspicuous versus cryptic colors, and colors associated with bird or mammal dispersal. Conspicuous colors are identified in the literature as colors that contrast with background foliage, for example, red, yellow, orange, whereas cryptic fruits are those that do not contrast with background foliage, for example, green, brown, based on a human trichromatic phenotype (Melin et al., 2008). We report that, in most cases, participants show a high degree of agreement on color categorization, but that there are discrepancies among participants, especially between speakers of different languages. Moreover, we find that reflectance spectra of the fruits classified to separate color categories by human observers show a high degree of overlap once processed through a nonhuman animal visual system, indicating that they do not correspond well to each other.

## 2 METHODS

We assembled a photographic database of 67 wild fruiting species from two sites in Madagascar (Ranomafana National Park, Ankarafantsika National Park) and one site in Uganda (Kibale National Park). Photographs were collected from the author's own photographic databases of fruits. For all fruits in the database, we had previously measured their reflectance in the field, relative to a Spectralon white reflectance standard (Labsphere, North Sutton, NH), using a Jaz portable spectrometer and a PX‐2 pulsed xenon lamp (Ocean Insight, Orlando, FL) emitting a D‐65 light source. The fruit scanning angle was fixed at 45° using a probe holder, and external light was blocked using thick black fabric. Each fruit was scanned 3–5 times, and the resulting spectrograms represent mean spectral reflectance. See Valenta et al. (2018) for detailed methods. The list of species is available at the supplementary materials.

Using freely available Google Forms software, we generated an online survey that had participants view a photograph of a single fruit species and select one of ten colors that best described the target fruit: red, orange, yellow, green, blue, purple, pink, brown, white, and black (online supplementary materials). Because the photographs often portrayed multiple fruits of the same species, we identified the target fruit with a white circle (Figure S1). Each target fruit and its associated color query was presented on a single page, allowing participants to focus on one target fruit at a time. We selected the ten color categories to coincide with manuscripts that rely on fruit color categorizations for their analyses (Janson, 1983; Onstein et al., 2020; Sinnott‐Armstrong et al., 2018). To collect data on additional variables that may influence color perception, we asked each participant to report their age, biological sex, native language, and whether they had any known color vision deficiency (either suspected or medically diagnosed). Because electronic devices can vary in their use of color and light, thereby changing the representation of a given image, we additionally requested information about the electronic device used to complete the survey (type, brand, and year). To exclude the possibility that variance in display types did not introduce substantial amounts of noise, we repeated all analyses (see below) on a subset of participants who reported using Apple iPhone models from 2017 onwards. These devices are equipped with OLED displays (“true black”) and are expected to provide highly comparable, if not identical, color displays. The results were practically identical to the results of entire dataset (Figure S2), which led us to conclude that differences in display types did not contribute a significant amount of noise. Therefore, to increase the sample size and linguistic diversity of our sample, we used the full dataset in all analyses. Informed consent was obtained from all participants, and the research was in accordance with relevant guidelines and regulations. All research was approved by the University of Florida Institutional Review Board for research on human subjects (IRB Protocol # 202,001,589).

We circulated the survey online, allowing for an opportunistic, snowball sampling technique, and collected responses between April 23, 2020 and May 7, 2020. We removed all responses by individuals under the age of 18, per the requirements of the ethics review board (IRB, University of Florida). To reduce the potential impact of charging and brightness settings, participants were asked to ensure their viewing device was plugged in and charging and to maximize their device's screen brightness. In total, we analyzed 786 survey responses, of which 20 participants self‐reported suspected or diagnosed color vision deficiencies (e.g., red‐green colorblindness). We included these individuals in all analyses under the assumption that many observers contributing to published reports of fruit color, especially among field assistants, may not report or even be aware of their color vision deficiencies. Additionally, the results are qualitatively identical even if they are excluded from the analyses.

To test whether color classifications among participants were significantly more consistent than expected by chance, we conducted a randomization test. Using the collected data, we first classified each fruit to a single color based on the plurality vote, that is, the color most commonly attributed to the fruit among all participants, and calculated the “consensus index”**—**the percentage of individuals that assigned a fruit species to the most commonly assigned color. We then simulated 999 randomized datasets that assumed colors were randomly assigned to each fruit. For each simulated dataset, we calculated the percentage of randomized responses that matched the color originally attributed to these fruits by a plurality of participants in the collected data. We then compared the collected classifications to the generated distribution to obtain a p‐value. We further used descriptive statistics to estimate the degree of discrepancy between participants. We calculated the percentage of misclassifications (classification of a fruit to a color different from the one determined by the plurality of participants) to determine whether any colors tended to be interrelated in their classifications (i.e., were fruits with a plurality of red classifications more likely to be misclassified as orange versus green). We then conducted two analyses that divided the ten colors to two major bins often used in ecological studies of fruit color: (a) conspicuous (defined in the literature as red, orange, yellow, pink) versus cryptic (green, blue, purple, brown, white, black) coloration, based on Onstein et al. (2020); and (b) colors related to bird dispersal syndromes (red, blue, purple, pink, white, black) versus mammal dispersal syndromes (orange, yellow, green, brown), following Janson (1983), and Sinnott‐Armstrong et al. (2018). We included these divisions in our analyses because many of the ten colors are often classified to the same functional bin, and thus disagreement between colors within one of these bins may be functionally meaningless, depending on the research question.

To assess whether speakers of different languages classified fruits to different colors, for each fruit, we identified the color selected by a plurality of participants in each of six languages for which we had at least 20 native speakers (English: 445 participants; Spanish: 47; French: 110; German: 66; Malagasy: 22; Portuguese: 21). Other languages reported as native languages among the participants were Malay, Afrikaans, Armenian, Bahasa, Bengali, Catalan, Chinese, Czech, Danish, Doteli, Dutch, Filipino, Greek, Gujarati, Hebrew, Hindi, Italian, Japanese, Malay, Persian, Polish, Russian, Telugu, and Vietnamese.

To assess whether human classifications are likely to be similarly classified by nonhuman observers, we used the reflectance data of all 67 fruit species included in the survey. We standardized the data by trimming the reflectance under and above the visual spectrum (400–700 nm), smoothed it using a running average with pavo: procspec (Maia et al., 2019), and converted the reflectance to relative amounts (i.e., standardizing the total reflectance across all samples). We then calculated the quantum catch for each photoreceptor for each fruit in two model organisms, representing two common visual systems: dichromatic mammals (dog) and tetrachromatic UV‐perceiving birds (average avian pigment sensitivity), assuming homogenous illumination, transmission, and background using pavo:vismodel. We then used pavo:coldist to calculate the chromatic distance between each pair of fruits in the dog and avian model systems. To test whether clusters are distinguishable, we used PERMANOVA on the quantum catch on each photoreceptor and 999 permutations. To visualize the results and examine to what degree human‐defined clusters are apparent in these two visual systems, we conducted a principal coordinate analysis (PCA) on the resulting distance matrices.

## 3 RESULTS

### 3.1 Agreement among human observers

**FIGURE 1:**
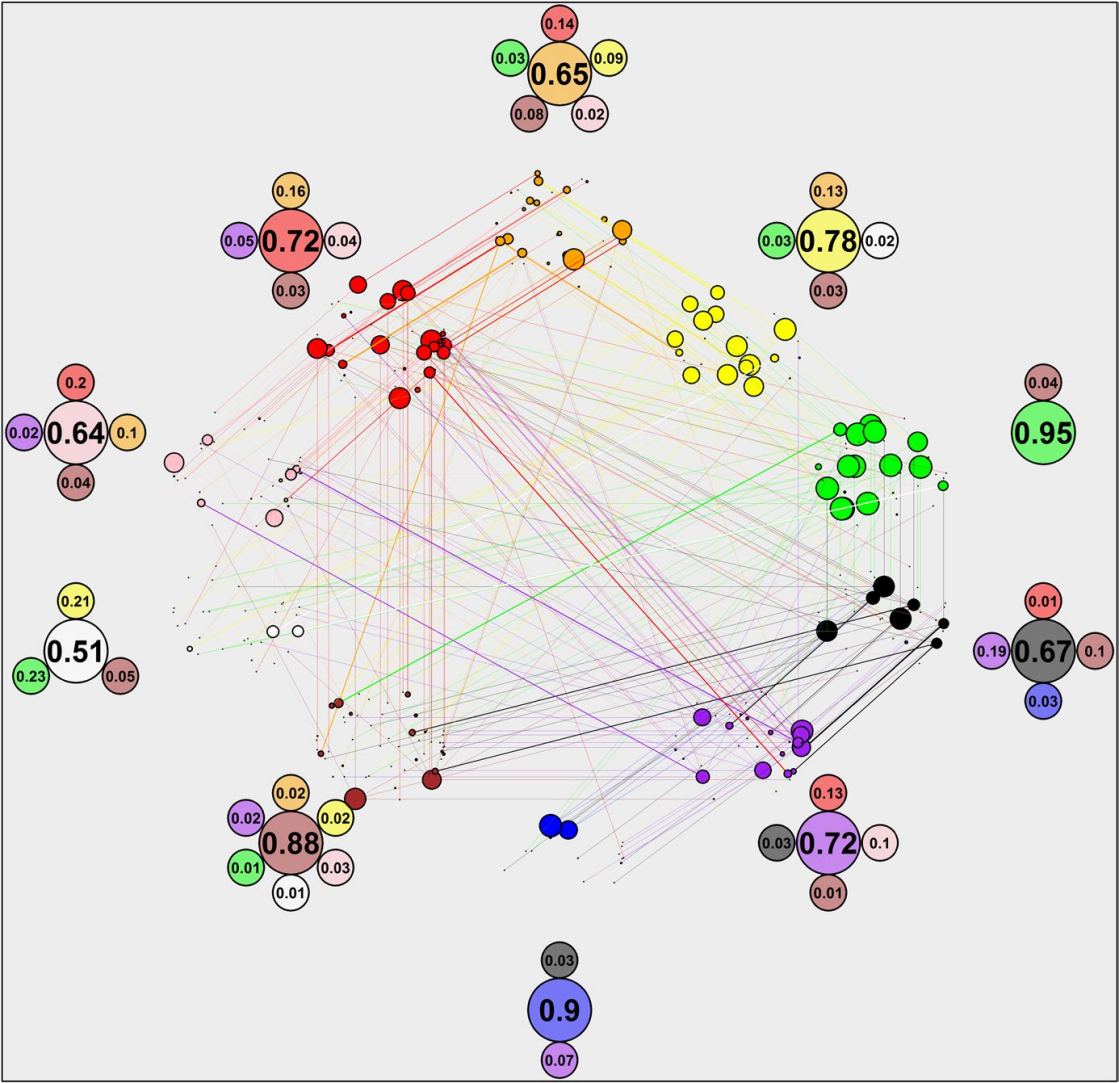
Degree of agreement among participants on fruit color classification. Inner circles represent raw data. Each inner circle is a fruit assigned by at least one participant to a certain color, and the circle size corresponds to the percentage of participants assigning a color to that fruit. Lines connect the most commonly assigned color for each fruit to other colors used to classify the same fruit. Distances between dots *within color* do not represent real differences in participant classification and are the result of random placement by the algorithm to avoid overlap between points. For example, a large red circle connected to a medium orange and small brown circle indicates that the fruit was classified primarily as red, but with a significant share of misclassifications to orange, and a small minority to brown. The outer section provides summary statistics for each color: The large circles give the percent of participants who agreed with the plurality opinion, and the smaller circles around show the breakdown of the misclassifications

Fruits were consistently assigned to the same color across survey participants (randomization tests, all ten colors: *p* < .01). This indicates that colors are not assigned randomly and that the majority of participants agreed on the color of each fruit. At the same time, classifications never reached true consensus: misclassifications (compared to the majority classification) were apparent in all color groups, with high degrees of disagreement over white, pink, orange, red, and purple fruits, as opposed to near consensus in brown, blue, and green fruits (Figure 1). Notably, fruits that were classified as white by half of the participant were classified as green or yellow by the other half.

**FIGURE 2:**
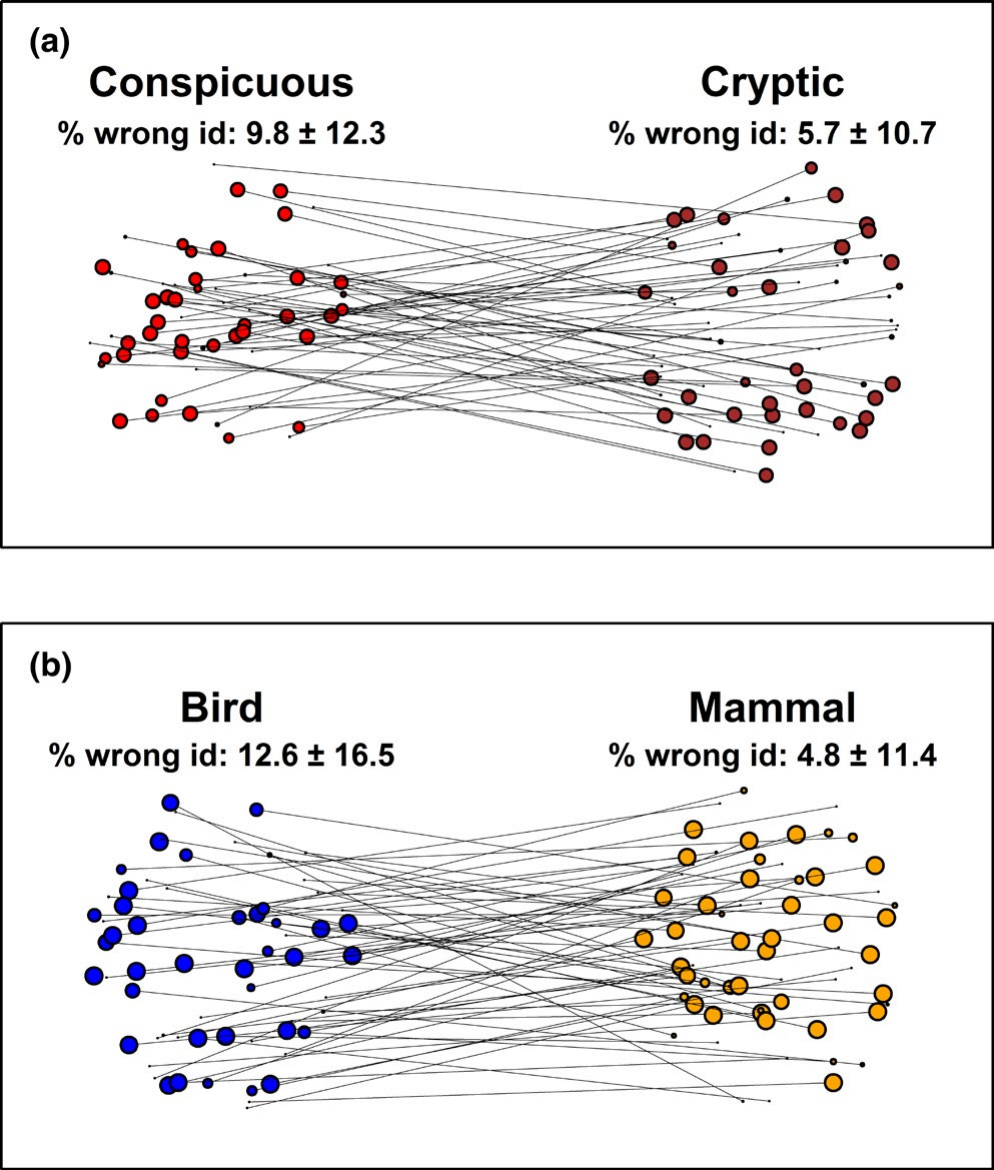
Classification of fruits to major bins: (a) conspicuous versus cryptic, and (b) bird versus mammal dispersal syndromes. Conspicuous: red, orange, yellow, pink. Cryptic: green, blue, purple, brown, white, black. Bird: red, blue, purple, pink, white, black. Mammal: orange, yellow, green, brown. Each circle is a fruit classified into either bin. Circle size corresponds to the share of participants who classified the fruit to the bin, and lines connect the same fruit if classified by at least some participants to the other bin. Location within a bin (e.g., “Bird”) is meaningless. Dots were jittered around the center of each category to visualize the variance in agreement among participants for each individual fruit

Fruits were also consistently assigned to the four bins (conspicuous versus cryptic; bird versus mammal), but with differing degrees of consistency (Figure 2). Fruits classified as conspicuous (red, orange, yellow, pink) versus cryptic (green, blue, purple, brown, white, black) were assigned to these bins by 90.2 ± 12.3% and 94.3 ± 10.7% of participants, respectively (Figure 2a). Fruit colors associated with bird dispersal (red, blue, purple, pink, white, black) versus mammal dispersal (orange, yellow, green, brown) were classified as such with an average agreement of 87.4% ± 16.5 and 95.2% ± 11.4, respectively (Figure 2b). However, disagreement among participants was not negligible, particularly for colors related to the bird dispersal syndrome, in which, on average, 12.6% ± 16.5 of participants classified fruits in such a way that they would be assigned to colors related to a mammalian dispersal syndrome.

Although native speakers of different languages showed a high degree of consistency, some clear discrepancies arose: In the six best‐represented languages in our sample, 22.3% of fruits were classified by a plurality of participants to at least two different colors (Figure S3). Classifications to color bins were more consistent across languages because misclassifications tended to include colors in the same bin as the color chosen by the plurality (e.g., purple and black). For 3% of fruits, speakers of different languages misclassified fruits in such a way as to alter their placement with respect to the conspicuous or cryptic bins. This type of misclassification occurred for 7.5% of fruits and their placement with respect to bird or mammal dispersal syndromes.

### 3.2 Agreement between human observers and animal visual systems

**FIGURE 3:**
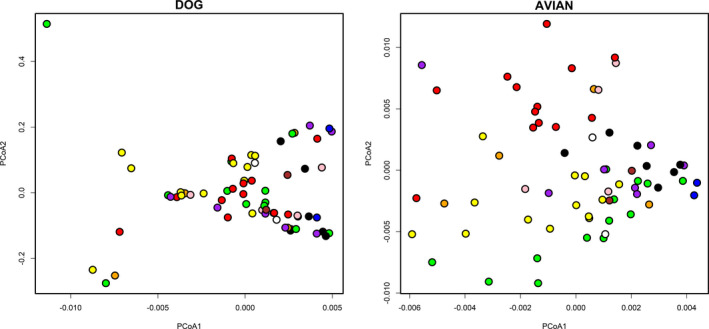
Chromatic distances between fruit in two visual systems. Principal coordinate analysis of chromatic distances based on quantum catch in dogs (dichromatic mammals) and an average UV‐perceiving avian. Each dot is a single fruit. Dot colors represent the human plurality classification

**FIGURE 4:**
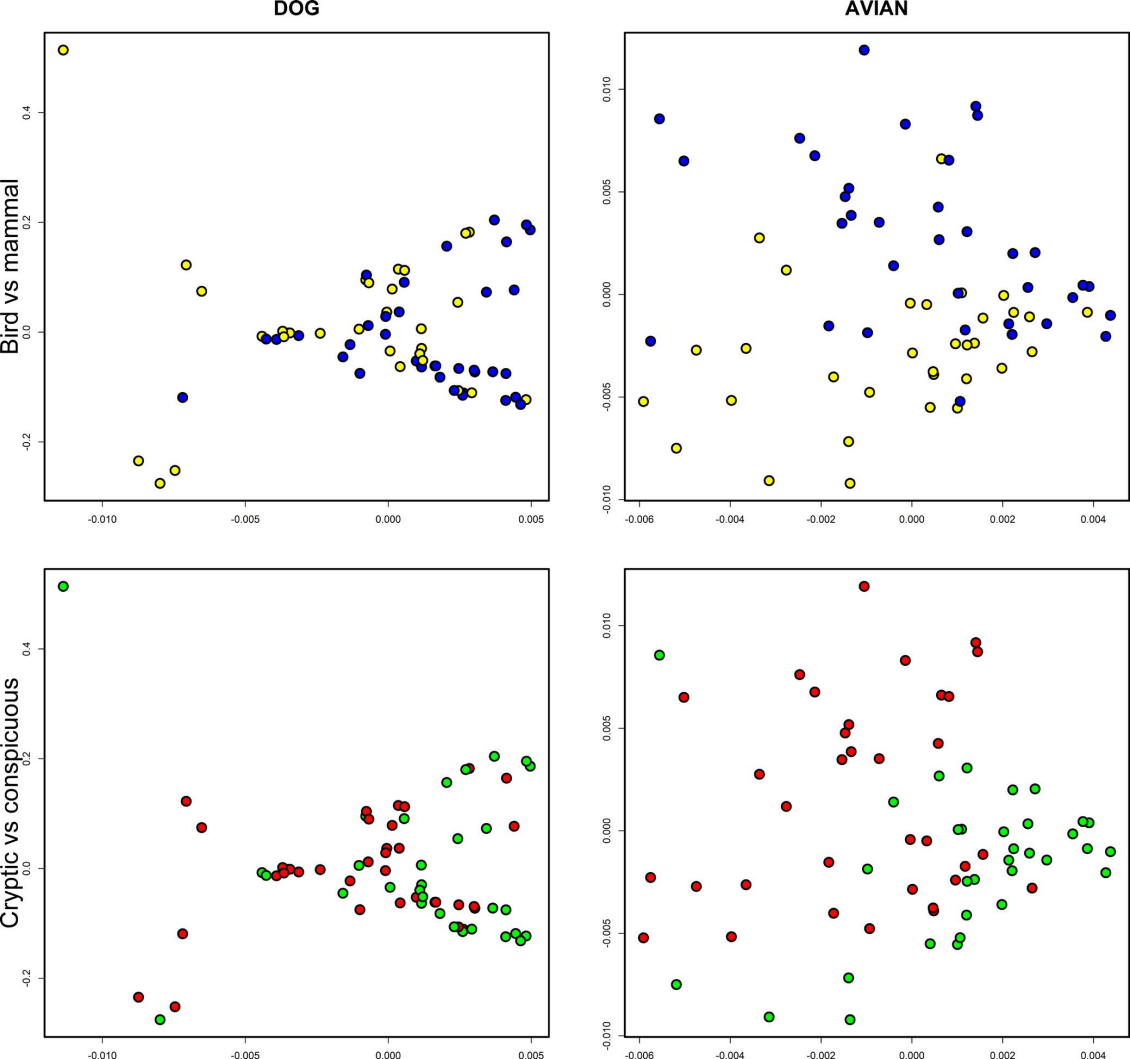
Chromatic distances of fruit reflectance in dog and avian visual systems between functional bins Principal component analysis similar to Figure 3. In all graphs, the x‐axis is PCoA1, and the y axis is PCoA2. Functional bins are classified based on commonly used classifications: bird (black, white, red, pink, purple, blue), mammal (green, brown, orange, yellow); cryptic (brown, black, green, blue, white, purple), conspicuous (red, orange, yellow, pink). In the top panel, blue dots represent colors binned to a bird dispersal syndrome, yellow dots represent colors binned to a mammal dispersal syndrome based on the plurality opinion. In the bottom panel, red dots represent conspicuous colors, and green dots represent cryptic colors, again based on the plurality opinion

Color classifications by humans showed partial agreement with the clustering patterns of chromatic distances based on the visual systems of a dichromatic mammal (dog) and a UV‐perceiving avian. In the avian visual system, some human‐classified color categories (e.g., red, green, yellow) formed coherent clusters while others (e.g., orange, pink, white) did not (Figure 3, Table 1). Functional bins (bird versus mammal, cryptic versus conspicuous) were also strongly distinguishable (Figure 4) (PERMANOVA, 999 permutations. *F* = 12.4, *p* < .001; *F* = 19.7, *p* < .001, respectively). Discrimination between human‐classified colors was lower in the dog visual system (Figures 3 and 4, Table 1), but differences between the major functional bins were still significant (PERMANOVA, 999 permutations. Bird versus mammal: *F* = 11.4, *p* = .004; cryptic versus conspicuous: *F* = 7.66, *p* = .01).

**TABLE 1:** *p* values from pairwise PERMANOVA tests in dog (upper triangle) and avian (lower triangle) visual systems

	Red	Orange	Yellow	Pink	White	Brown	Green	Black	Blue	Purple
Red	1	.731	.112	.316	.491	.347	.747	**.001**	**.026**	.189
Orange	**.013**	1	.543	.342	.733333	.533333	.921	**.021**	.133333	.31
Yellow	**.001**	.342	1	**.045**	.121	.06	.294	**.001**	**.006**	**.014**
Pink	**.01**	.723	.054	1	.933333	.933333	.316	.104	.266667	.903
White	**.013**	.666667	.359	.533333	1	.666667	.553	**.028**	.333333	.792
Brown	**.008**	.4	.105	.333333	1	1	.428	.075	.333333	.95
Green	**.001**	**.011**	**.003**	**.004**	.331	.261	1	**.008**	.054	.18
Black	**.001**	.071	**.002**	.071	.391	.47	**.007**	1	.22	.191
Blue	**.009**	.133333	**.013**	.066667	.333333	.333333	**.01**	.075	1	.225
Purple	**.005**	.453	**.049**	.854	.93	.862	**.002**	.565	.198	1

Note

Lower triangle**—**avian. Upper triangle**—**dog. *p* Values lower than .05 are in bold.

## 4 DISCUSSION

Our goals were to identify whether human observers consistently classify fruits to the same colors and to assess the degree to which these classifications reflect how nonhuman observers may view the same fruit. Overall, we found that there was a high degree of consistency in color categorizations across survey participants, indicating that, in the human visible spectrum, color assessments made by human observers are generally consistent. However, there was some interesting variation among color classifications. For some colors, for example, green, there was a very high level of agreement, whereas for other colors, for example, white, categorization was less consistent.

Although fruits were consistently categorized to the same color, subsequent binning of those colors into ecologically relevant categories, as was done in previous studies (Onstein et al., 2020; Sinnott‐Armstrong et al., 2018), revealed interesting variation. In particular, human assessments of fruit colors related to a bird dispersal syndrome were not fully consistent: Across all four bins, the greatest discrepancy resulted from participants classifying fruits in such a way that they would be categorized with mammal dispersal colors, whereas the fruit's plurality color placed them in the bird dispersal bin. Perhaps unsurprisingly, this indicates that, as mammals, humans may be less reliable at identifying color signals that are associated with bird dispersal (e.g., white), compared to signals associated with mammal dispersal (e.g., green). In addition, across different languages, discrepancies were also greater after fruits were assigned to dispersal syndromes. Thus, although subjective single color categorizations might be consistent, their subsequent interpretation may introduce a non‐negligible amount of noise.

Comparison of human color classifications and the quantum catch of the reflectance data of the 67 species in a dichromatic mammal and a tetrachromatic bird revealed both the validity of human classifications and its limitations. Among birds, whose color discrimination capacities surpass humans’, some of the more common colors (red, green, yellow) formed clear clusters that were statistically distinguishable from most other human‐classified colors. At the same time, some were not, and all showed substantial overlap with other colors, indicating that a high degree of consistency in human color perception. For example, fruits classified as green by humans can, in the eyes of birds, be closer to fruits classified as white, brown, or blue by humans. Nonetheless, the fact that many colors were distinguishable, and that the main functional bins were also clearly distinct, is an indication that human classification, while not noise‐less, is a reasonable proxy for chromatic variance.

An additional consideration is that participants in the survey viewed fruit photographs on different devices and screens, and the resulting variation in hue, saturation, and chromaticity could have influenced our results. While we cannot exclude that this introduced some noise, we believe that it has little effect on our results for several reasons. First, this noise is expected to reduce interparticipant agreement, thus making the analysis even more conservative and strengthening our conclusion that participants show a high degree of agreement. Second, our analysis of a subset of the participants who used very similar devices reproduces the results, thereby indicating that display types did not play a major role in subjective color classification. Third, potential variation introduced by differences in viewing devices likely pales in comparison to that introduced by the myriad light and viewing conditions in the field, particularly in tropical forests where our fruit samples were taken (Endler, 1993; Yoshimura & Yamashita, 2012). Furthermore, many field studies rely on observers to categorize the color of ripe fruits and to identify which fruits are ripe among a possible array of fruits at different developmental stages (Brodie, 2017; Burns et al., 2009; Lu et al., 2019; Onstein et al., 2019; Sinnott‐Armstrong et al., 2018). For example, in the field, an observer may identify a species that, from their perspective, transforms from an unripe green state, through yellow, to orange, to red, to black. The observer may decide that the orange or red stage represents the color at ripeness, whereas the black stage represents overripe fruit. However, a different observer may categorize the black stage as indicating the color of ripeness for the same species. Here, we removed variation that might be introduced in field‐based studies, by eliminating the potential for observers to misclassify different stages of ripeness by indicating the exact fruit or fruits to categorize.

Taken together, our results indicate that although fruit color categorization is largely consistent, differences in language, and the binning of fruit colors into larger, ecological categories (e.g., bird dispersal syndromes) can introduce non‐negligible variation. More critically, discrete color categories are strongly affected by either human color vision capacities or the arbitrary division of the spectrum, yielding a classification pattern that is not fully reflective of the chromatic distances perceived by nonhuman observers. This is likely to be particularly true in systems with bird dispersal‐dominated flora, for example, temperate forests, where UV reflectance is probably high. As such, our results show that studies that utilize subjective color observations are reliable to the degree that the variance between human observers is likely to be low. However, studies relying on subjective color classifications should exercise caution since human color categorizations can be affected by physiological, linguistic, and/or cultural biases and do not correspond well to the colors perceived by animals with different color vision phenotypes.

## CONFLICT OF INTEREST

KV, SLB, and ON declare that publication of this manuscript will enhance their CV and publication statistics and thus improve their academic prestige and employment status. Y‐DJ has no conflict of interest to declare.

## AUTHOR CONTRIBUTION

**Kim Valenta:** Conceptualization (equal); Data curation (equal); Investigation (equal); Methodology (equal); Project administration (equal); Writing‐original draft (equal); Writing‐review & editing (equal). **Sally Bornbusch:** Conceptualization (equal); Data curation (equal); Investigation (equal); Methodology (equal); Project administration (equal); Resources (equal); Visualization (equal); Writing‐original draft (equal); Writing‐review & editing (equal). **Yan‐Daniel Jacques:** Data curation (equal); Investigation (equal); Methodology (equal); Project administration (equal); Writing‐original draft (equal); Writing‐review & editing (equal). **Omer Nevo:** Conceptualization (equal); Data curation (equal); Formal analysis (equal); Validation (equal); Visualization (equal); Writing‐original draft (equal); Writing‐review & editing (equal).

## Supporting information

Appendix S1

## Data Availability

All data are available via Dryad at: Valenta, Kim; Bornbusch, Sally; Jacques, Yan‐Daniel; Nevo, Omer (2022), In the eye of the beholder: Is color classification consistent among human observers?, Dryad, Dataset, https://doi.org/10.5061/dryad.nk98sf7tq
