# Supplementary material for: In the eye of the beholder: Is color classification consistent among human observers?
Source: Ecol Evol. 2021 Sep 14;11(20):13875–83. doi: 10.1002/ece3.8093 (PMC8525178; doi:10.1002/ece3.8093)

Supplementary materials

**Valenta et al. 2021. In the eye of the beholder: Is color classification consistent among human observers?**

**Survey preface:**

Thank you for agreeing to participate in this short experiment.

You will be shown 67 photographs of fruit and be asked to pick JUST ONE of a list of colors provided that best describes the dominant color of the fruit/fruits circled in the picture. Fruits may appear to have multiple colors, please choose ONE of the following colors that is most representative of the circles fruit/fruits:

Red

Orange

Yellow

Green

Blue

Purple

Pink

Brown

White

Black

THERE ARE NO WRONG ANSWERS.

Please note that some of the images are blurry or pixelated. Please overlook issues with image quality and do your best to focus on the color.

Before we get started, there are few questions to answer. The answers to these questions will in no way change anything about the test.

BEFORE YOU BEGIN, please do the following:

1. Make sure your electronic device is plugged in and charging
2. Maximize your electronic device's screen brightness

If you have any questions or concerns, please contact the principal investigator at: kimvalenta[at]ufl.edu

**Survey Questions:**

What was your biological sex at birth? (choose one)

- Male
- Female
- Prefer not to say

What is your age? (enter a digit)

What is your first language? (enter name of language)

What is your second language? (enter name of language)

What is your third language? (enter name of language)

Are you aware of, or have you been diagnosed with any color vision deficiencies, for example, color blindness? (choose one)

- Yes
- No

If you answered "Yes" to the previous question, please describe. (enter information)

Please select the type of device you are using to take this survey. (choose one)

- Laptop computer
- Desktop computer
- Tablet
- Mobile phone

Please select the manufacturer of the device you are using to take this survey?

- Acer
- Alcatel
- Apple
- Asus
- Dell
- Google
- HP
- HTC
- Huawei
- Lenovo
- LG
- Microsoft
- Motorola
- Nokia
- Panasonic
- Philips
- Samsung
- Sony
- Toshiba
- ZTE
- Other

To the best of your knowledge, in what year was your device manufactured?

- 2010
- 2011
- 2012
- 2013
- 2014
- 2015
- 2016
- 2017
- 2018
- 2019
- 2020

For each of the next 67 photos of fruit, answer the following question:

What color best represents the circled fruit/fruits?

- red
- orange
- yellow
- green
- blue
- purple
- pink
- brown
- white
- black

**Figure S1.** Photos presented in the survey. Species names included here were not in the survey.

| 1 | 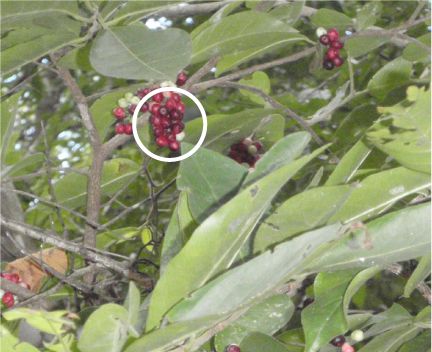 | *Antidesma petiolare* |
| --- | --- | --- |
| 2 | 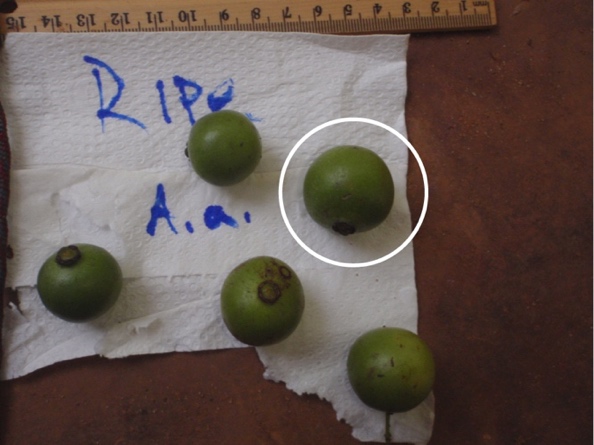 | *Asterotrichilia asterotricha* |
| 3 | 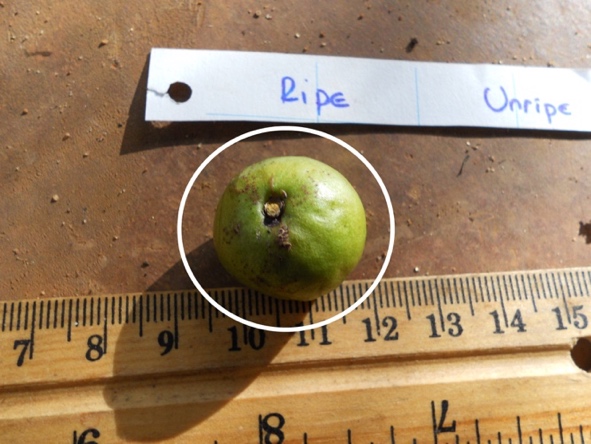 | *Asterotrichilia spp.* |
| 4 | 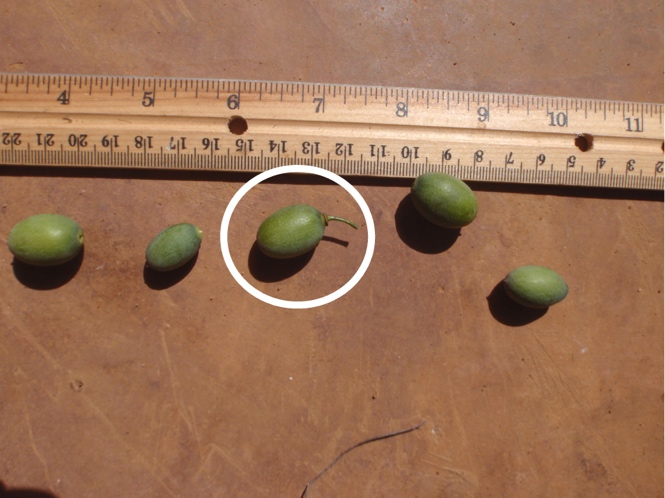 | *Berchemia discolor* |

| 5 | 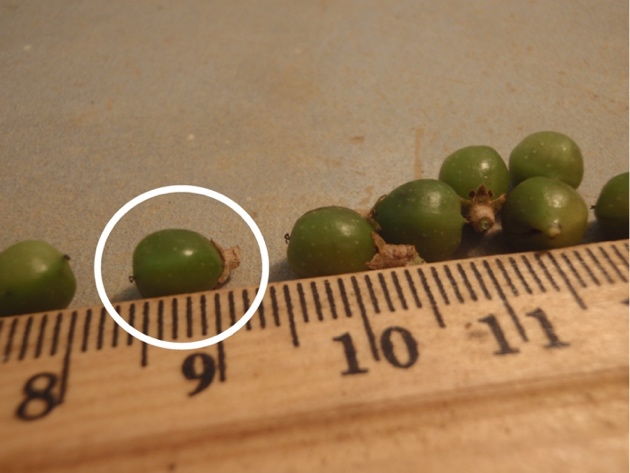 | *Bridelia pervileana* |
| --- | --- | --- |
| 6 | 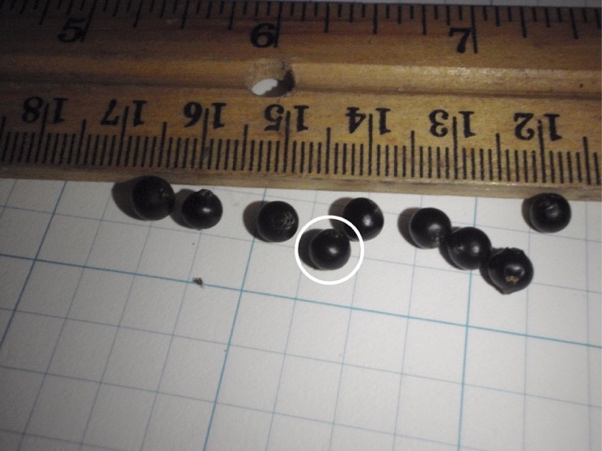 | *Croton spp.* |
| 7 | 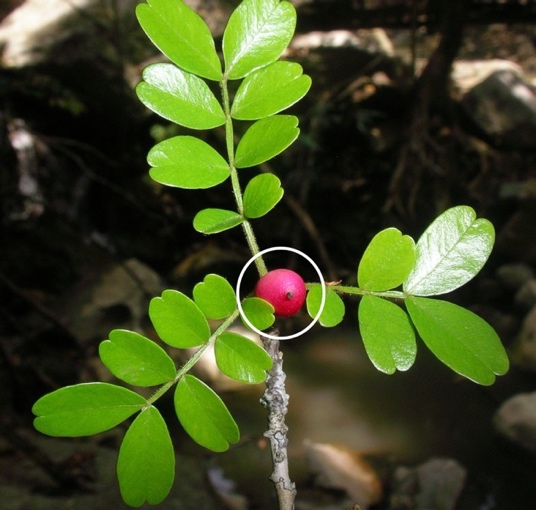 | *Dorotoxylon choux* |
| 8 | 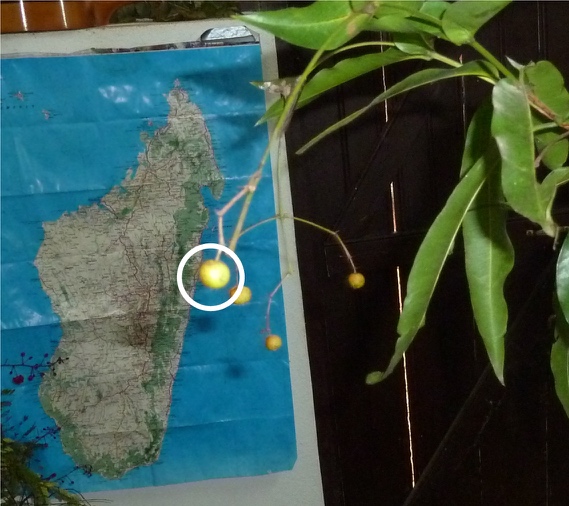 | *Elaeocarpus subserrata* |

| 9 | 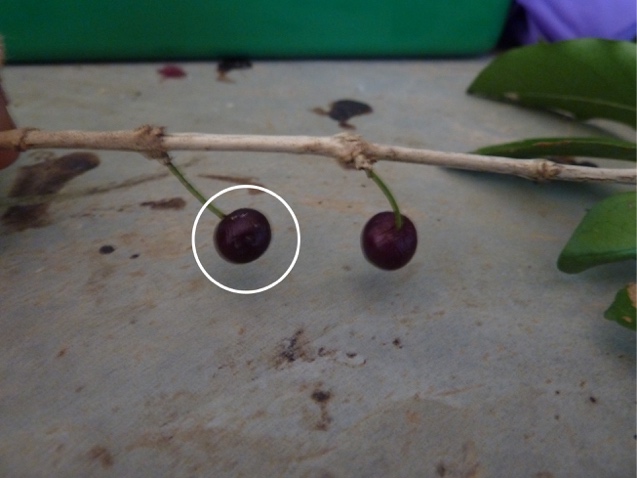 | *Empogona ovalifolia* |
| --- | --- | --- |
| 10 | 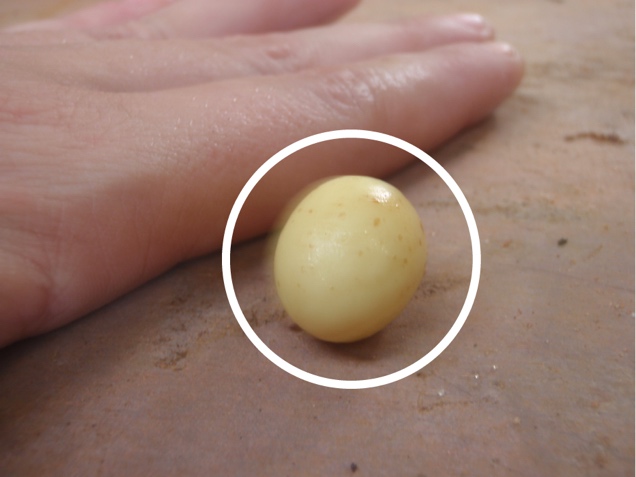 | *Garcinia arenicola* |
| 11 | 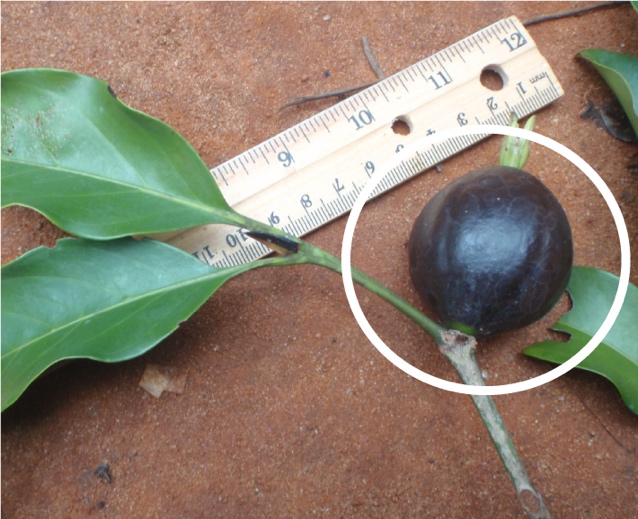 | *Gardenia rutenburgiana* |
| 12 | 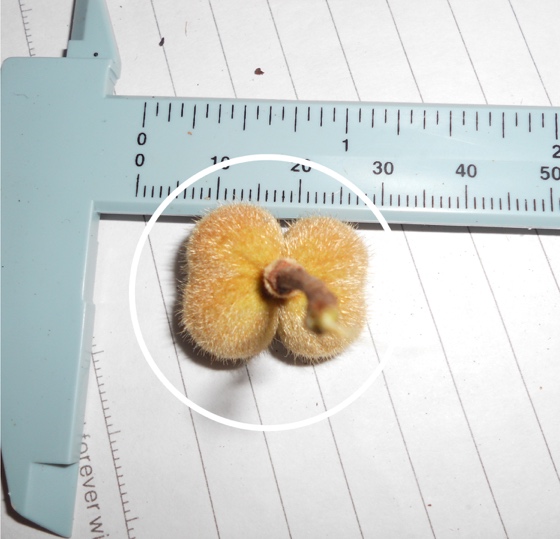 | *Grewia madagascariensis* |

| 13 | 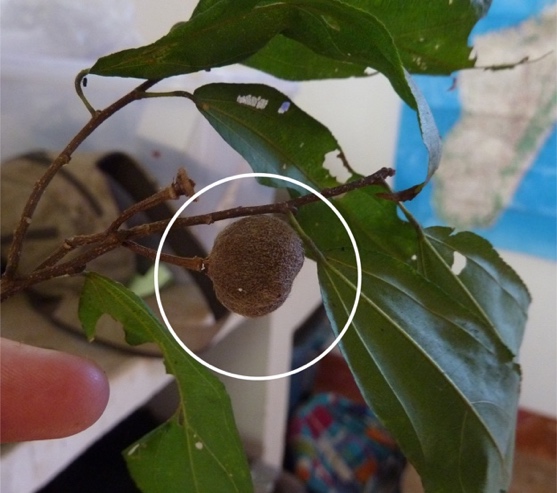 | *Grewia spp.* |
| --- | --- | --- |
| 14 | 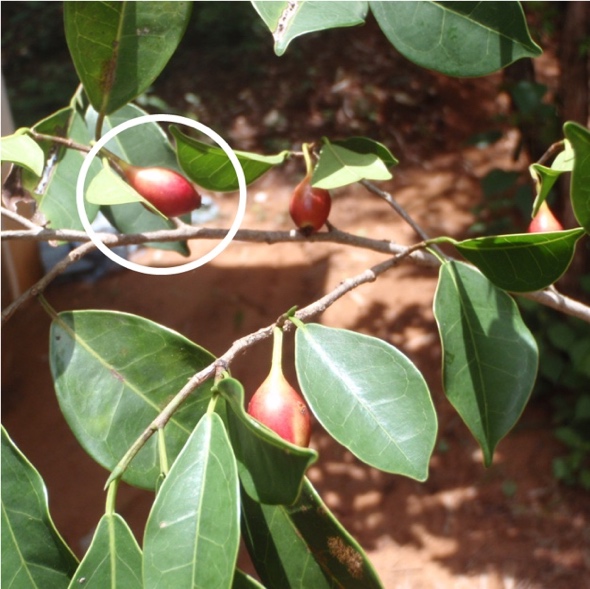 | *Noronhia spp.* |
| 15 | 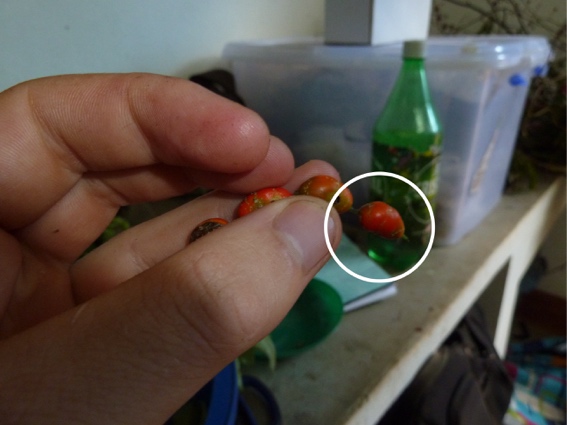 | *Petchia spp.* |
| 16 | 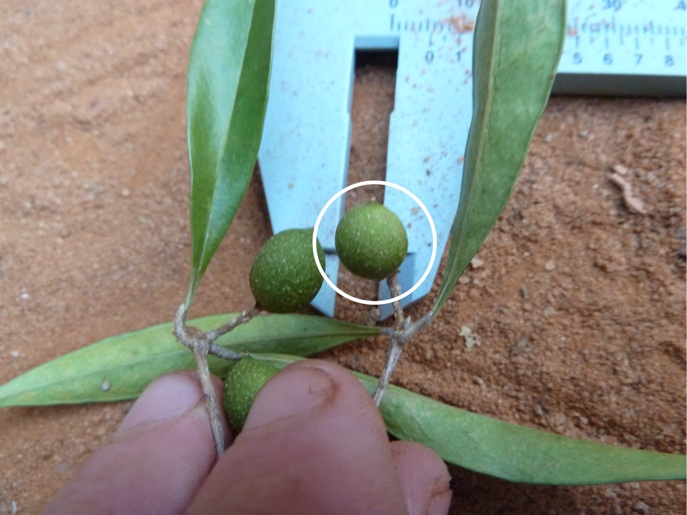 | *Salvadora angustifolia* |

| 17 | 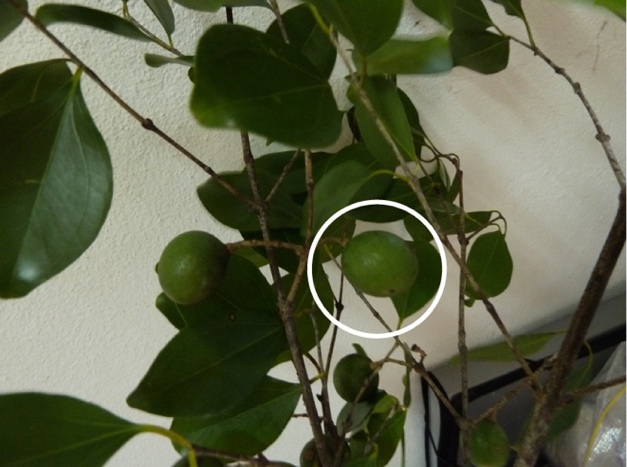 | *Strychnos decussata* |
| --- | --- | --- |
| 18 | 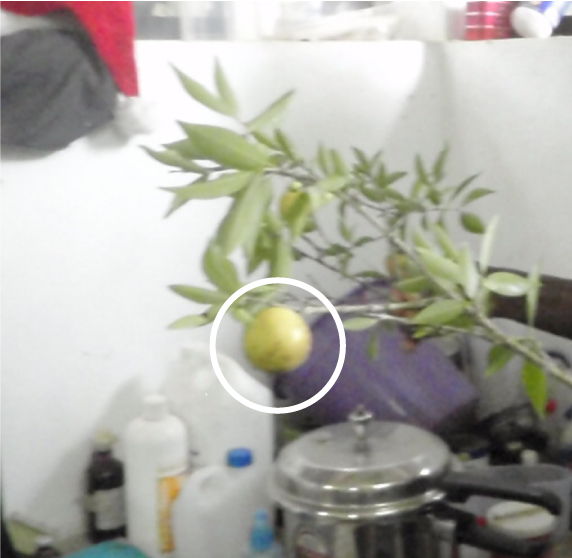 | *Strychnos madagascariensis* |
| 19 | 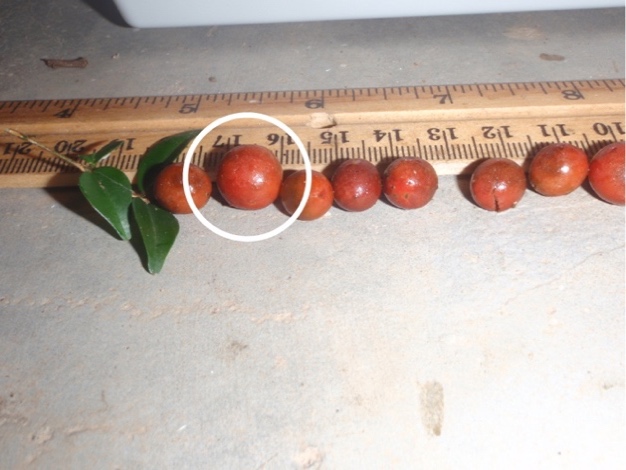 | *Strychnos myrtoides* |
| 20 | 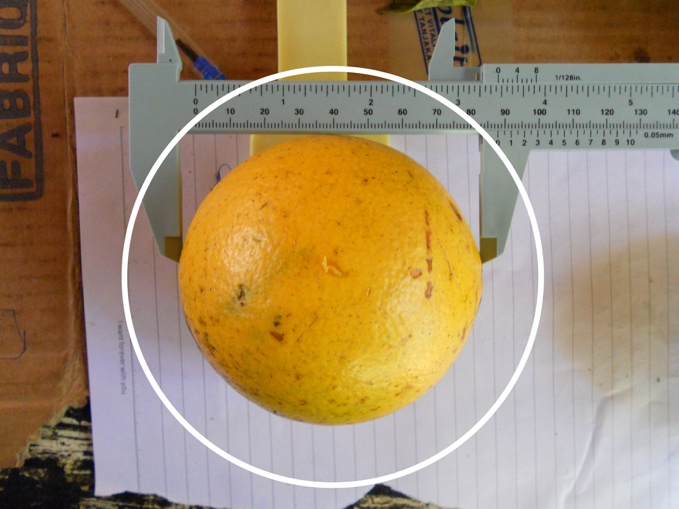 | *Strychnos spinosa* |

| 21 | 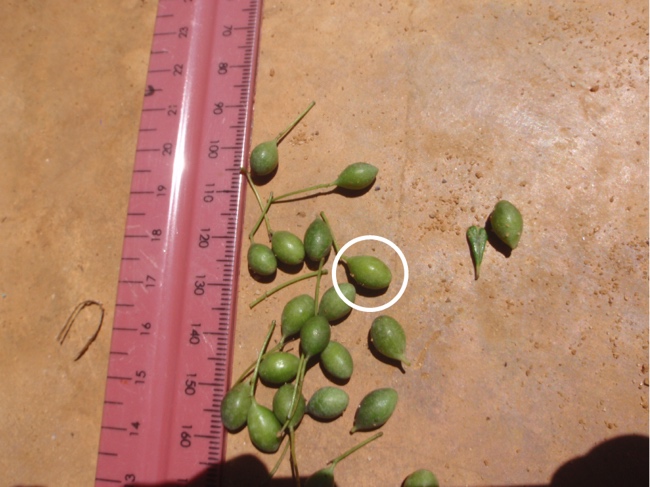 | *Terminalia tropophylla* |
| --- | --- | --- |
| 22 | 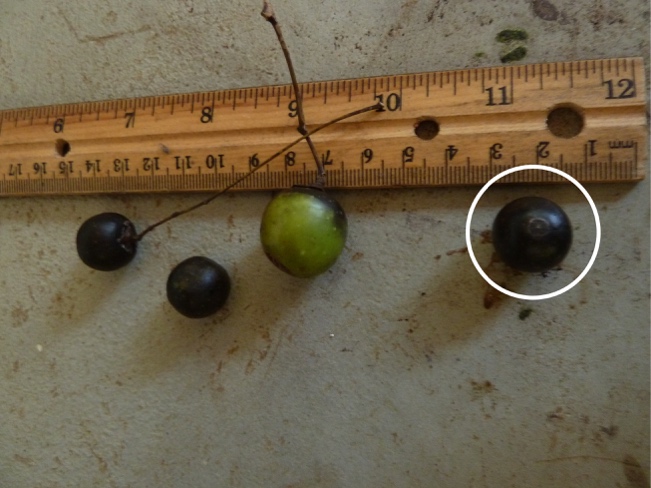 | *Vitex spp.* |
| 23 | 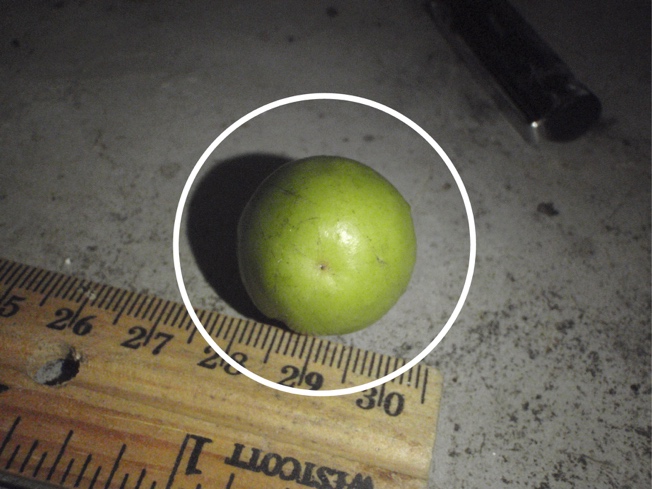 | *Ziziphus spp.* |
| 24 | 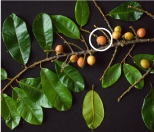 | *Aningeria altissima* |

| 25 | 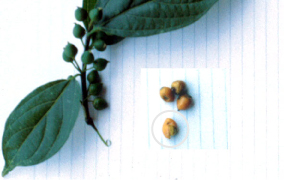 | *Celtis durandii* |
| --- | --- | --- |
| 26 | 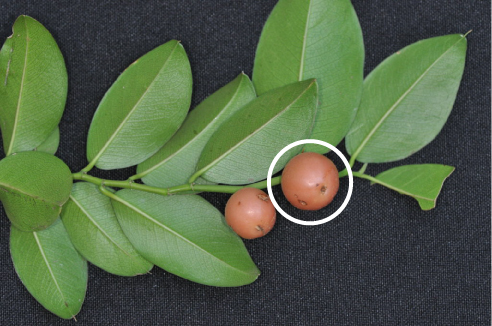 | *Chatacme aristata* |
| 27 | 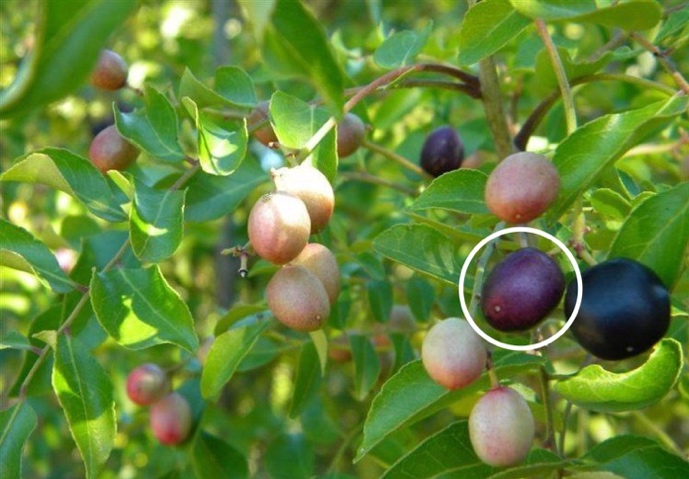 | *Clausena anisata* |
| 28 | 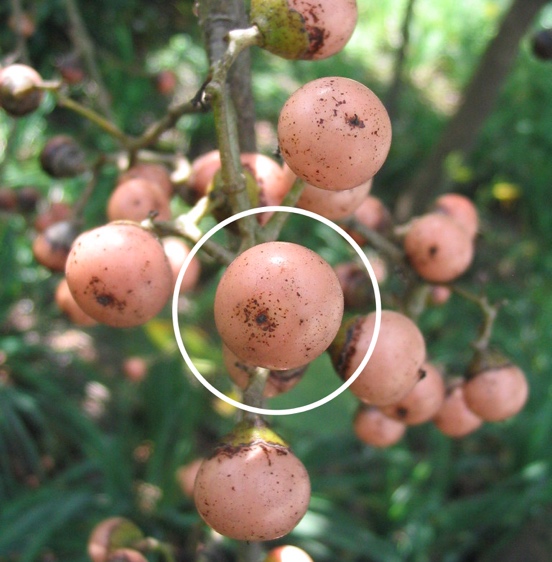 | *Cordia abyssinica* |

| 29 | 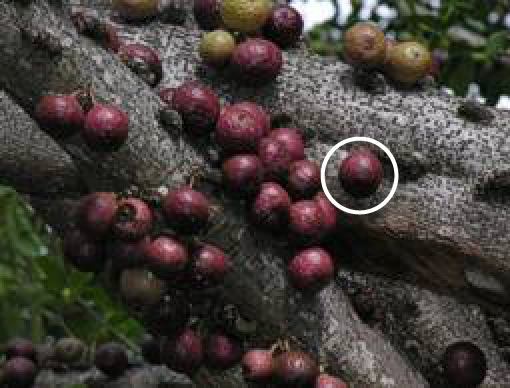 | *Ficus brchylepsis* |
| --- | --- | --- |
| 30 | 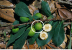 | *Ficus cyathistipula* |
| 31 | 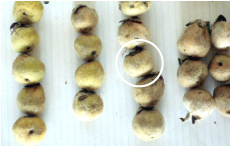 | *Ficus daweii* |
| 32 | 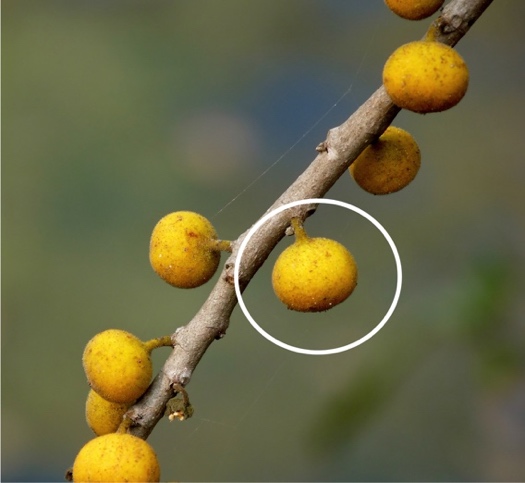 | *Ficus exasperata* |

| 33 | 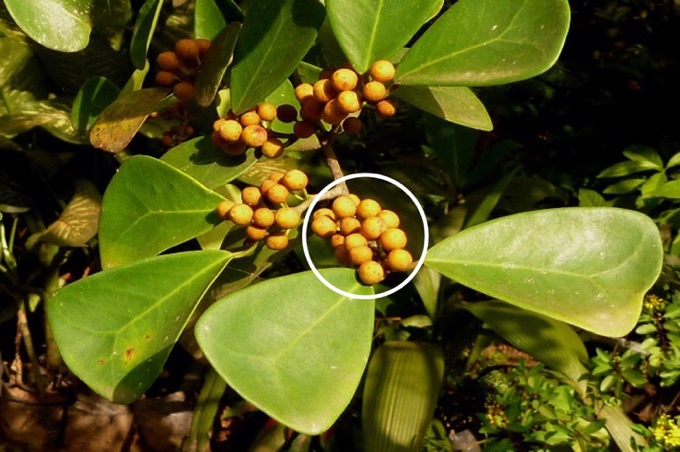 | *Ficus natalensis* |
| --- | --- | --- |
| 34 | 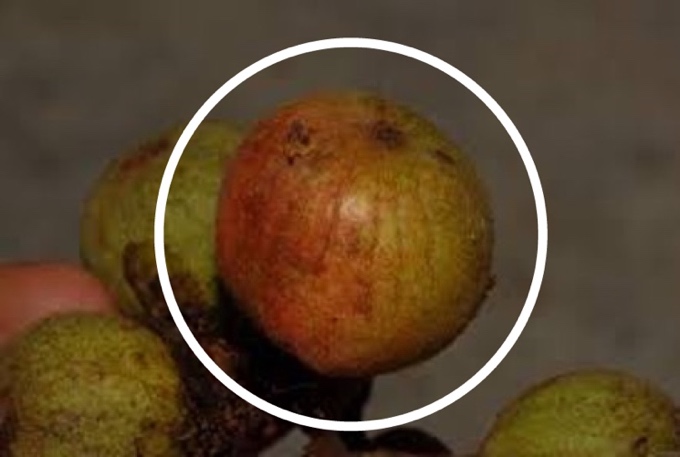 | *Ficus vallis* |
| 35 | 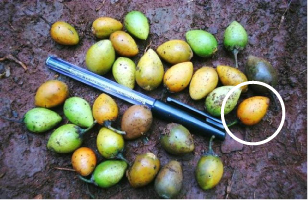 | *Mimusops bashaweii* |
| 36 | 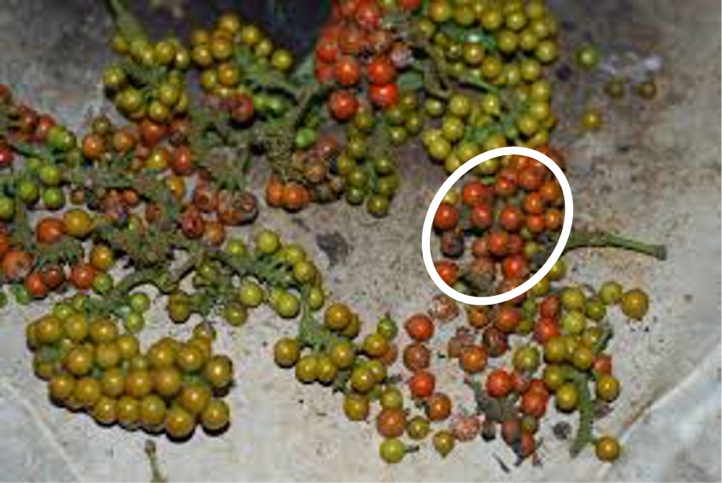 | *Piper guineense* |

| 37 | 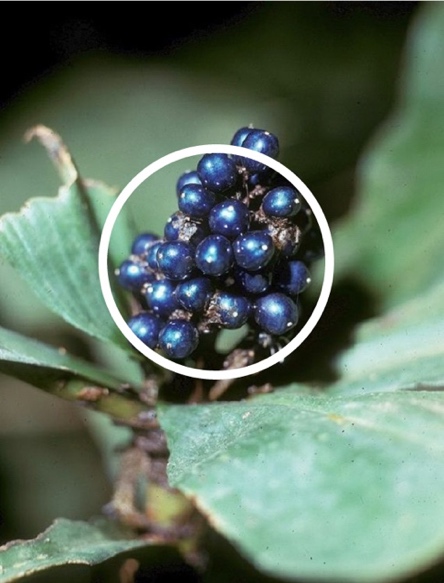 | *Pollia spp.* |
| --- | --- | --- |
| 38 | 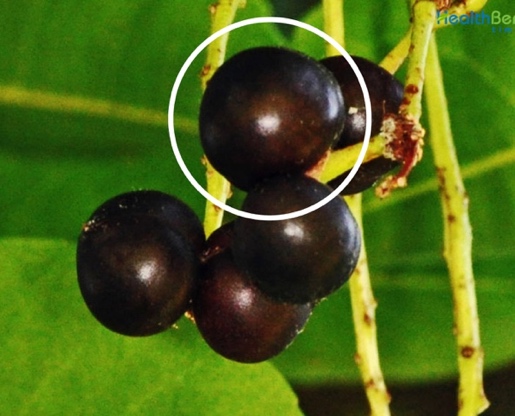 | *Prunus africana* |
| 39 | 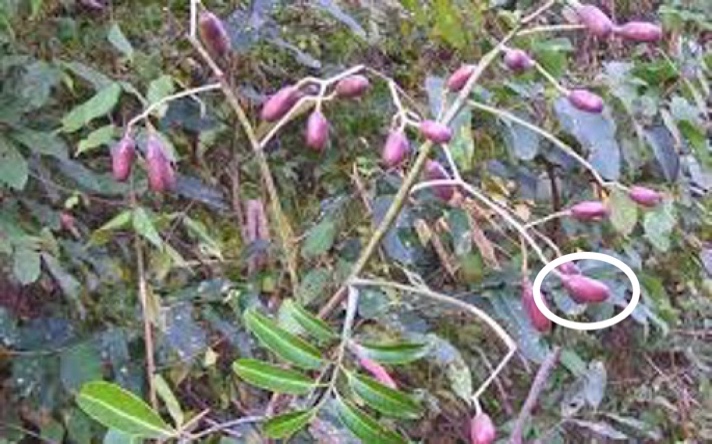 | *Pseudospondias microcarpa* |
| 40 | 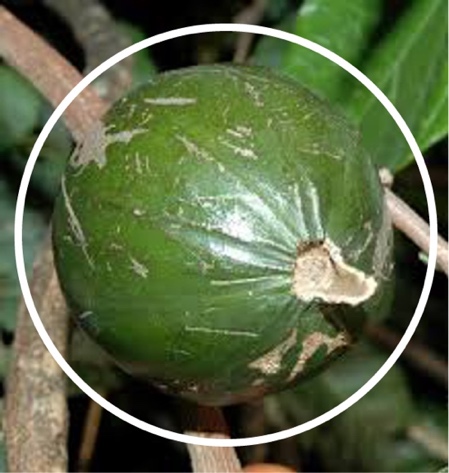 | *Rothmania urcelliformis* |

| 41 | 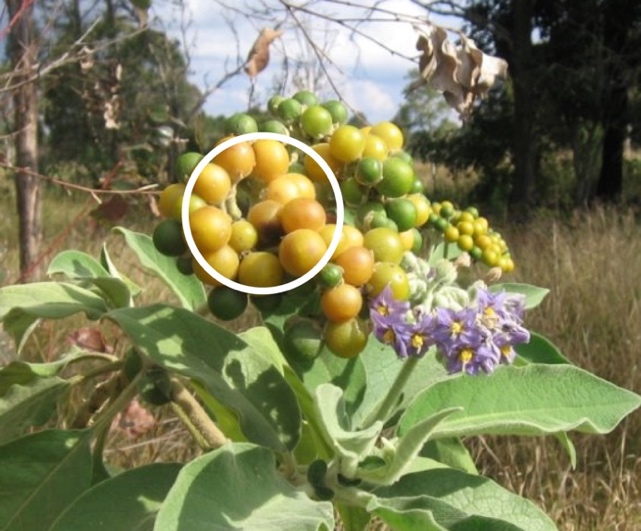 | *Solanum mauritanium* |
| --- | --- | --- |
| 42 | 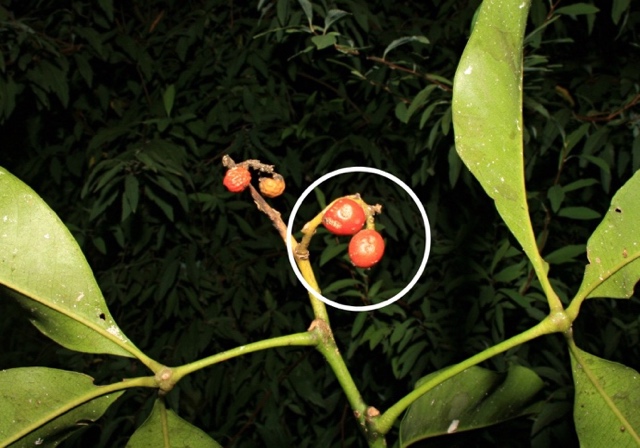 | *Teclea nobilis* |
| 43 | 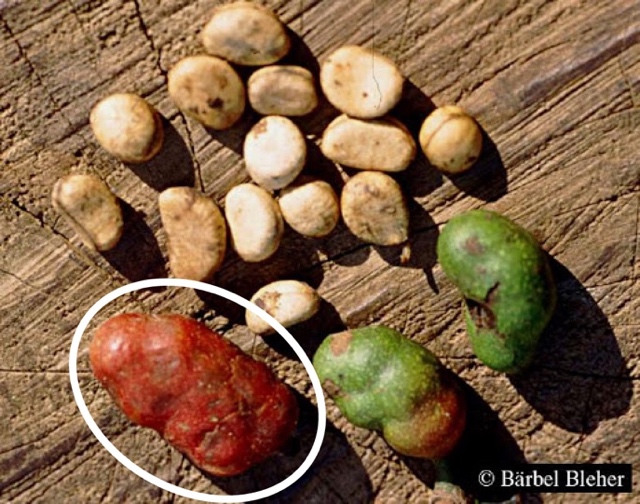 | *Uvariopsis congensis* |
| 44 | 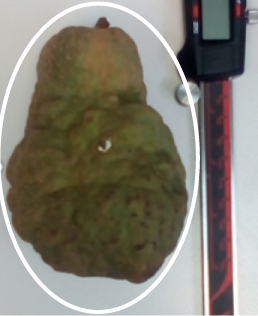 | *Agelae pentagyna* |

| 45 | 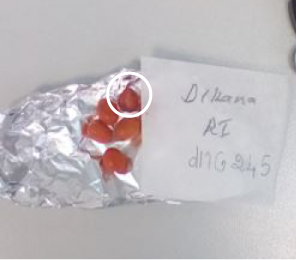 | *Allophylus arboreus* |
| --- | --- | --- |
| 46 | 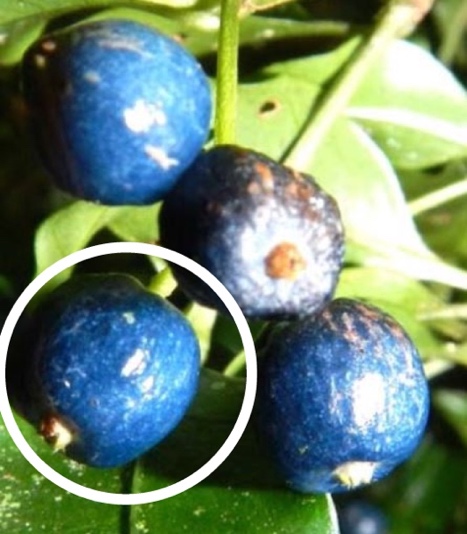 | *Chassalie ternifolia* |
| 47 | 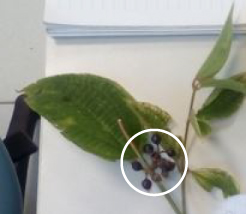 | *Clidemia hirta* |
| 48 | 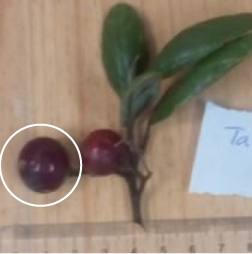 | *Cryptocarya crassifolia* |

| 49 | 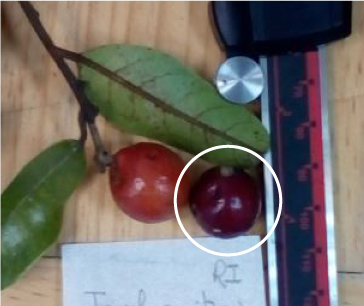 | *Cryptocarya spp.* |
| --- | --- | --- |
| 50 | 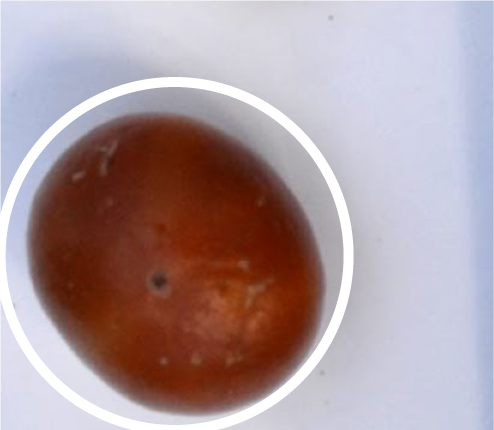 | *Dracaena reflexa* |
| 51 | 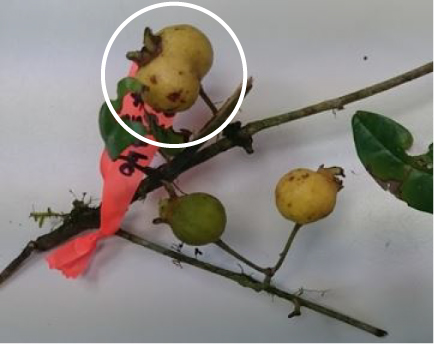 | *Eugenia spp.* |
| 52 | 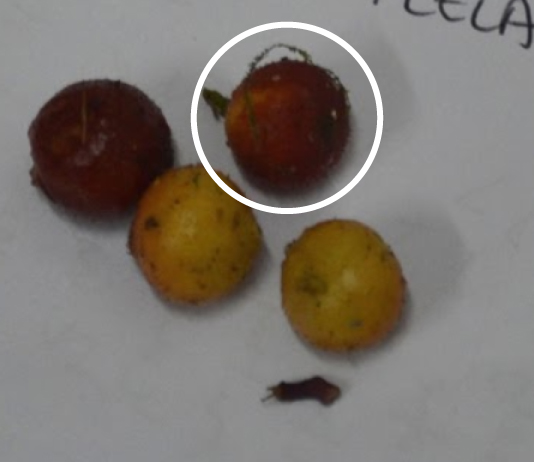 | *Ficus politoria* |
| 53 | 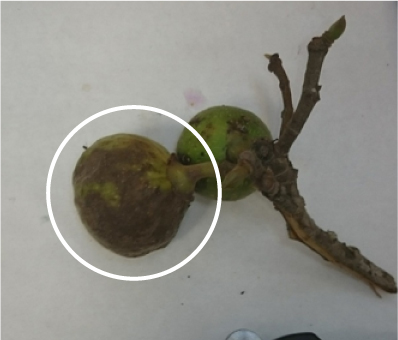 | *Ficus tilliifolia* |
| 54 | 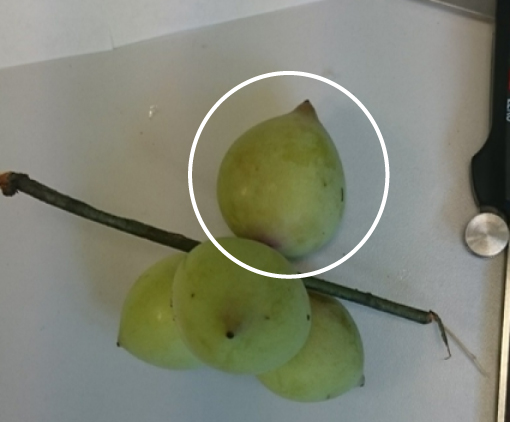 | *Garcinia spp.* |
| 55 | 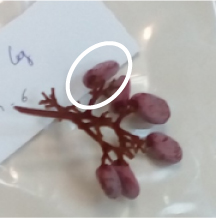 | *Micronychia macrophylla* |
| 56 | 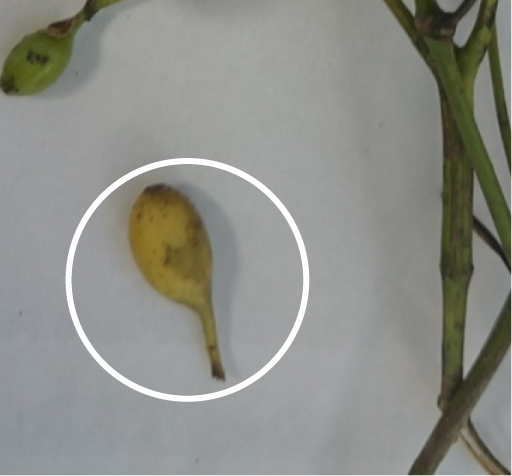 | *Mussaenda arcuata* |
| 57 | 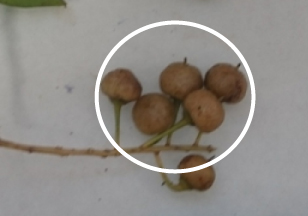 | *Oncostemum botryoides* |
| 58 | 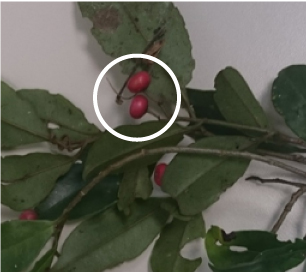 | *Oncostemum nervosum* |
| 59 | 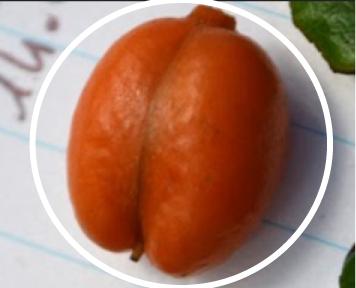 | *Pittosporum pachyphyllum* |
| 60 | 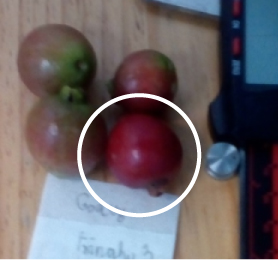 | *Psidium cattleianum* |
| 61 | 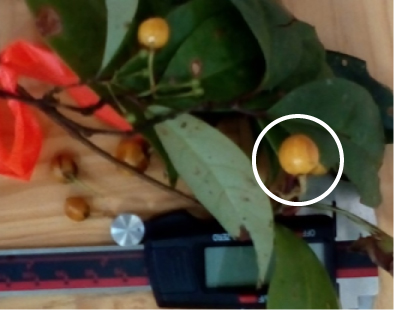 | *Psorospermum androsaemifolium* |
| 62 | 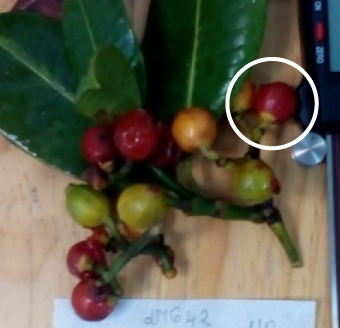 | *Psychotria spp.* |
| 63 | 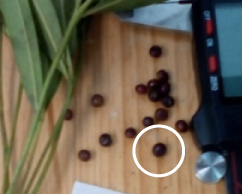 | *Schefflera spp.* |
| 64 | 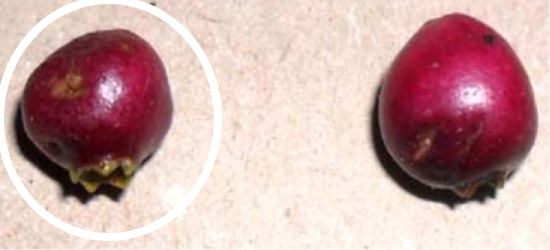 | *Syzygium emirnese* |
| 65 | 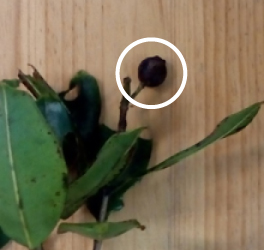 | *Syzygium parkeri* |
| 66 | 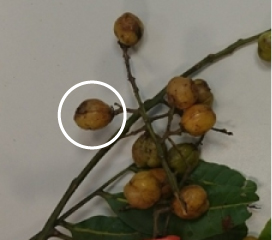 | *Tina striata* |
| 67 | 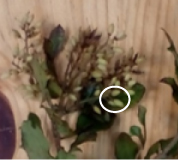 | *Weinmannia rutenbergii* |

**Figure S2**. Degree of agreement among participants on fruit color classification, showing only OLED display users. Inner circles represent raw data. Each inner circle is a fruit assigned by at least one participant to a certain color, and the circle size corresponds to the percentage of participants assigning a color to that fruit. Lines connect the most commonly assigned color for each fruit to other colors used to classify the same fruit. Distances between dots *within color* do not represent real difference in participant classification and are the result of random placement by the algorithm to avoid overlap between points. For example, a large red circle connected to a medium orange and small brown circle indicates that the fruit was classified primarily as red, but with a significant share of misclassifications to orange, and a small minority to brown. The outer section provides summary statistics for each color: the large circles gives the percent of participants who agreed with the plurality opinion, and the smaller circles around give the breakdown of the misclassifications. These results using only one type of device are highly similar to the results using the entire dataset (Figure 1).


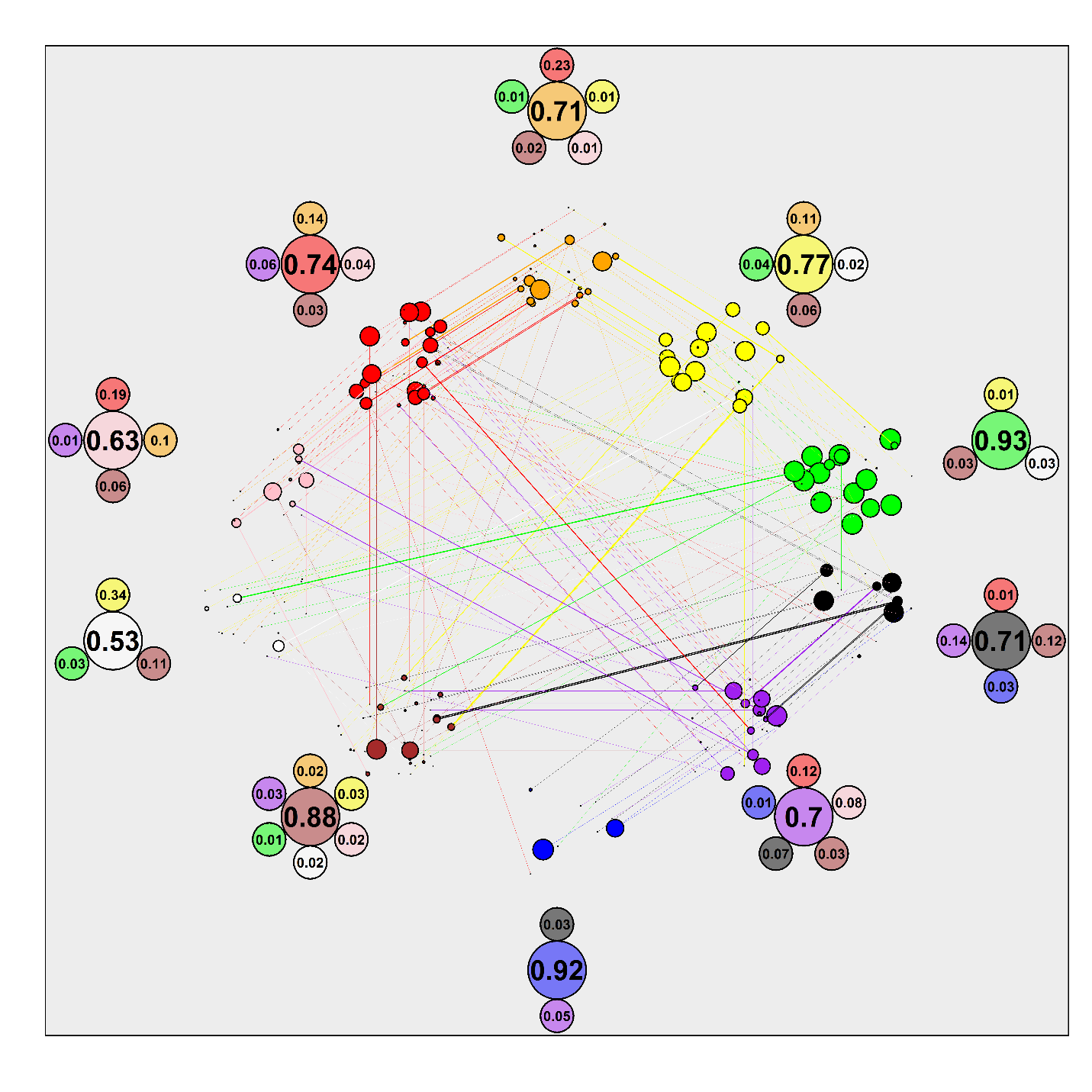


**Figure S3.** The interaction between native language and color categorization. Each column is a language, representing one of the six most common native languages in our survey sample: English, Spanish, French, German, Malagasy, and Portuguese. Each row represents an individual fruit species. For each fruit/language combination the corresponding pie chart shows the categorization of each fruit color, organized into the ten color categories. The background color indicates the plurality vote within a language – when background colors are different from others in a row, native speakers of different languages disagreed in their overall classification. Numbers of each row correspond to the numbers of each species shown in the survey (see species list above).


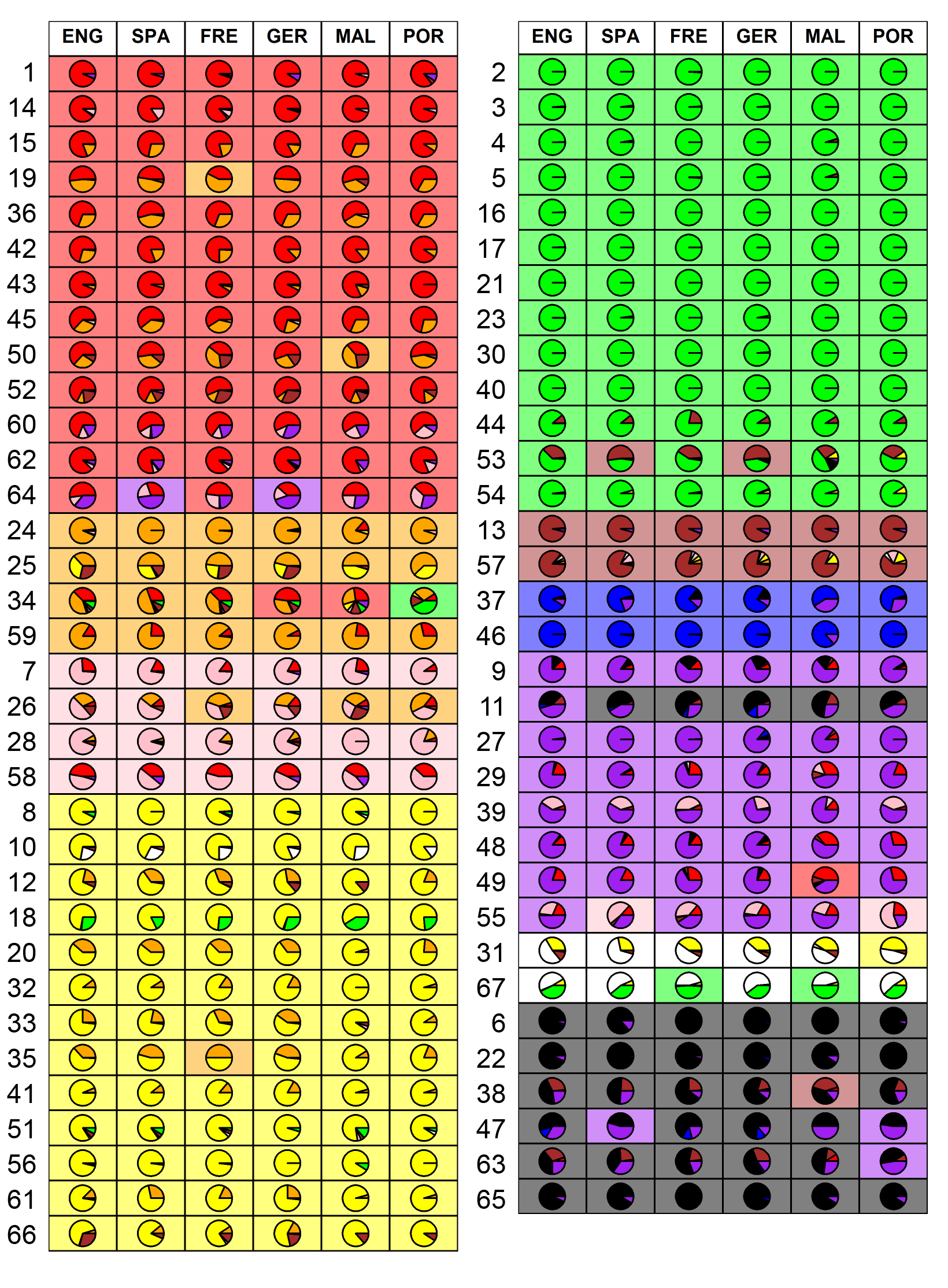

Supplement: Supplementary file 1 — Appendix S1 [file ECE3-11-13875-s001.docx]
